# Primary central nervous system tumors in Sergipe, Brazil: descriptive epidemiology between 2010 and 2018

**DOI:** 10.1590/0004-282X-ANP-2020-0151

**Published:** 2021-06-30

**Authors:** Bárbara Loiola SANTOS, Arthur Maynart Pereira OLIVEIRA, Hélio Araújo OLIVEIRA, Robson Luis Oliveira de AMORIM

**Affiliations:** 1 Universidade Federal de Sergipe, Departamento de Medicina de Lagarto, Lagarto SE, Brazil. Universidade Federal de Sergipe Departamento de Medicina de Lagarto Lagarto SE Brazil; 2 Universidade Federal de Sergipe, Departamento de Medicina, Aracaju SE, Brazil. Universidade Federal de Sergipe Departamento de Medicina Aracaju SE Brazil; 3 Fundação de Beneficência Hospital de Cirurgia, Serviço de Neurocirurgia, Aracaju SE, Brazil. Fundação de Beneficência Hospital de Cirurgia Serviço de Neurocirurgia Aracaju SE Brazil; 4 Universidade Federal do Amazonas, Programa de Pós-Graduação em Ciências da Saúde, Manaus AM, Brazil. Universidade Federal do Amazonas Programa de Pós-Graduação em Ciências da Saúde Manaus AM Brazil

**Keywords:** Brain Neoplasms, Epidemiology, Prevalence, Neoplasias Encefálicas, Epidemiologia, Prevalência

## Abstract

**Background:**

Central nervous system (CNS) tumors are a heterogeneous group with high morbidity and mortality.

**Objectives:**

To describe the epidemiology of primary CNS tumors diagnosed in the state of Sergipe from 2010 to 2018.

**Methods:**

We evaluated histopathological and immunohistochemical reports on primary CNS tumors diagnosed in Sergipe, Brazil, between 2010 and 2018 and collected data regarding age, sex, location, World Health Organization (WHO) classification and histology.

**Results:**

Altogether, 861 primary CNS tumors were found. Tumors in brain locations occurred most frequently (50.8%; n=437). The neoplasms observed were most prevalent in the age range 45‒54 years (20.4%; n=176). Grade I tumors occurred most frequently, corresponding to 38.8% of the cases (n=38) in the age group of 0‒14 years, and 44.6% (n=340) in the population ≥15 years old. Between 0 and 14 years of age, other astrocytic tumors were the most prevalent (29.6%; n=29). In the age group between 15 and 34, gliomas were the most frequent (32.7%; n=54). Meningiomas predominated in the age group of 35 years and above, comprising 47.5% of cases (n=206) in the 35‒74 age group; and 61.2% (n=30) among patients over 75 years old.

**Conclusion:**

The epidemiology of primary CNS tumors in Sergipe between 2010 and 2018 is consistent with data in other current studies on the subject. Studies on the epidemiological evolution of these entities in Sergipe are needed.

## RESUMO

**Introdução:**

Os tumores do sistema nervoso central (SNC) são um grupo heterogêneo de entidades que apresenta significativa morbimortalidade.

**Objetivos:**

O presente estudo visa à descrição epidemiológica dos tumores primários do SNC com diagnóstico histopatológico no estado de Sergipe, Brasil, entre 2010 e 2018.

**Métodos:**

Foram avaliados laudos histopatológicos e imuno-histoquímicos de tumores primários do SNC, diagnosticados entre 2010 e 2018, no estado de Sergipe. Os dados coletados foram descritos de acordo com as variáveis de idade, sexo, localização, classificação da Organização Mundial da Saúde (OMS) e tipo histológico.

**Resultados:**

Foram encontrados 861 tumores primários do SNC. A localização cerebral foi a mais frequente (50,8%; n=437). As neoplasias observadas prevaleceram na faixa etária de 45 a 54 anos (20,4%; n=176). Os tumores grau I foram os mais frequentes, correspondendo a 38,8% dos casos (n=38) na faixa etária de 0‒14 anos e 44,6% (n=340) na população a partir de 15 anos de idade. Na faixa etária entre 0‒14 anos, o grupo histológico dos outros tumores astrocíticos foi o mais prevalente (29,6%; n=29). Na população entre 15‒34 anos, os gliomas foram os mais frequentes (32,7%; n=54). Os meningiomas predominaram nas faixas etárias a partir de 35 anos, com 47,5% dos casos (n=206) entre 35‒74 anos; e 61,2% (n=30) nos pacientes acima de 75 anos de idade.

**Conclusão:**

A descrição epidemiológica dos tumores primários do SNC em Sergipe, entre 2010 e 2018, é condizente com outros trabalhos atuais sobre o tema. Estudos voltados à evolução epidemiológica dessas entidades em Sergipe são necessários.

## INTRODUCTION

Disordered proliferation of brain cells originates tumors, which may be malignant or non-malignant^1^^,^^2^^,^^3^. In Brazil, it has been estimated that 11,090 new cases of malignant central nervous system (CNS) tumors will occur in 2020. Except for non-melanoma skin cancer, CNS neoplasms correspond to the tenth most prevalent type of cancer among women in Brazil, and are the ninth most frequent among men and women in the northeastern region of Brazil^4^. Because of the location, unpleasant neurological deterioration may be seen in cases of both malignant and non-malignant CNS tumors. Moreover, these neoplasms are responsible for significant mortality in the population^4^^,^^5^^,^^6^^,^^7^.

The World Health Organization (WHO) published a new update on the classification of tumors of the central nervous system in 2016. In this, their classification came to incorporate molecular characteristics, in addition to the histological aspects of tumor entities. The new perspective has an impact on diagnosing tumor types since it enhances discrimination between subtypes resembling histopathological categories and therefore optimizes the accuracy of diagnosis^8^^,^^9^^,^^10^.

Because of the heterogeneity of these tumors and the different methods used in studies, their precise epidemiological description is complex^11^^,^^12^^,^^13^. Moreover, many sites do not have a non-malignant tumor registration system. This limits the analysis on the distribution and characterization of these entities in the population^14^.

Comparative studies have shown increasing incidence of CNS tumors over the years^15^^,^^16^^,^^17^. This may be occurring through increased exposure to possible risk factors such as high-dose irradiation or genetic syndromes, or through improved access to screening imaging tests^18^^,^^19^^,^^20^. Furthermore, it has been suggested in some studies that the increasing use of wireless devices and cordless phones are risk factors for the development of CNS tumors^21^^,^^22^.

In Sergipe, a state in northeastern Brazil, few studies have evaluated the epidemiology of CNS tumors. This state does not have a notification system for benign CNS tumors, which highlights the importance of epidemiological studies based on the histopathological diagnosis of these entities. In Aracaju, a city located in Sergipe, the population-based cancer registry (RCBP) possesses a high-quality database, that supports national ratings for the incidence of primary malignant CNS tumors. This formed a reference point for parallels with the findings of the present study.

The objective of this study was to characterize the CNS primary tumors in Sergipe between 2010 and 2018. Through these data, we described the current panorama of distribution of these entities, with the aim of contributing to knowledge of the epidemiology of these tumors.

## METHODS

### Study design

This was an observational and descriptive retrospective longitudinal study in which histopathological reports on patients who underwent removal of primary CNS tumors in the state of Sergipe, Brazil between January 2010 and December 2018 were analyzed. The reports were collected from the database laboratories in Sergipe that agreed to participate in the study.

### Inclusion criteria

All patients who underwent surgery to treat primary CNS tumors in the state of Sergipe, between January 2010 and December 2018, and whose histopathological material was analyzed, were included in this study. Patients were enrolled regardless of their place of residence since their addresses were not available from the laboratories that attended to these cases.

### Exclusion criteria

We excluded patients whose histopathological reports were inconclusive, along with cases of metastasis. Among the histopathological reports, 52 ​​cases were found to be inconclusive or contained incomplete information, and were therefore excluded from this study.

### Data collection technique and instruments

The data-gathering period was between November 2018 and February 2019. After the methodology of the current project had been presented to the Research Ethics Committee of the Federal University of Sergipe and approval had been granted, data collection at the laboratories that agreed to participate started.

Five anatomopathological laboratories located in Aracaju, Sergipe, are registered in the National Register of Healthcare Establishments (CNES). Four out of these five were responsible for all of the anatomopathological reports on brain surgeries in Sergipe, and all of them agreed to participate in this current research.

The data analysis followed the 2016 WHO classification for stratification of CNS tumors, in which the tumors are divided into four groups, according to increasing degrees of severity, ranging from grade I to IV. In 2016, an updated classification was published, incorporating molecular characteristics to define tumor entities^10^. We used this updated classification for CNS tumors, but no molecular analysis had been performed in most cases. Therefore, given the small number of cases with molecular analysis, it was decided not to describe molecular data in this current study. After data collection, we analyzed the prevalence of tumors and their epidemiological characteristics according to the variables of age, sex and location, in accordance with the categories of the International Classification of Diseases, 10^th^ edition (ICD-10), WHO grade and histological tumor type.

### Ethical considerations

This study was approved by the Research Ethics Committee of the Health Campus of the Federal University of Sergipe (UFS). No consent form was applied because no personal identification of the patients was used. Nor was there any analysis of medical records or contact with any patient. Confidentiality of information regarding healthcare service user identification and anonymity in future publication of results will remain assured.

### Statistical data analysis

The data were systematized, analyzed and statistically tested using the Statistical Package for the Social Sciences (SPSS) software version 20.0 and the R software version 3.5.0. The variables were described through absolute and relative frequencies, medians, arithmetic means and standard deviations. After descriptive analysis, it was investigated whether the data followed normal distribution of probability, using the Kolmogorov-Smirnov test. The results of interest were tested using the nonparametric Mann-Whitney test.

## RESULTS

### Epidemiology of primary CNS tumors in the state of Sergipe between 2010 and 2018

Altogether, 861 primary CNS tumors were found in Sergipe between 2010 and 2018, after applying the study exclusion criteria. Out of the total, 56.9% (n=490) were in females and 43.1% (n=371) were in males. Overall, neuroepithelial tumors accounted for 50.2% (n=433) of the cases. Gliomas occurred most frequently, comprising 64.2% (n=278) of these entities and 32.3% (n=278) of the entire sample. Glioblastoma was the most common type of glioma, corresponding to 63.7% (n=177) of the glioma cases. Meningiomas were the second most prevalent tumor, present in 29.6% (n=255) of the sample. The meningothelial and transitional histological types of meningiomas accounted for 40.3% (n=103) and 25.4% (n=65) of the cases, respectively (Table 1). Meningiomas were the most common tumors in females, whereas among males, gliomas occurred most frequently (Table 1).

**Table 1.** Distribution of primary central nervous system tumors according to histological groups, Sergipe, Brazil, 2010‒2018.

Histological groups	Males		Females		Total	
	n	%	n	%	n	%
Gliomas	144	51.8	134	48.2	278	32.3
Meningiomas	73	28.6	182	71.3	255	29.6
Medulloblastomas	31	51.7	29	48.3	60	7.0
Ependymal tumors	21	50.0	21	50.0	42	4.9
Other astrocytic tumors	24	48.9	25	51.0	49	5.7
Schwannomas and neurofibromas	45	52.9	40	47.1	85	9.9
Tumors of the pineal region	2	50.0	2	50.0	4	0.5
Hemangiomas and hemangioblastomas	8	44.4	10	55.6	18	2.1
Craniopharyngiomas	2	33.4	4	66.6	6	0.7
Pituitary adenomas	14	31.8	30	68.2	44	5.1
Neuronal and mixed neuronal-glial gliomas	6	31.6	13	68.4	19	2.2
Germ cell tumors	2	100	-	-	2	0.2

The histological groups were also evaluated according to age groups. In the population from 0 to 14 years of age, 98 tumors were found. The group of "other astrocytic tumors" was the most prevalent, comprising 29.6% (n=29) of these entities. This group was followed by medulloblastomas and ependymal tumors, with 27.6% (n=27) and 14.3% (n=14) of the cases for this age group, respectively (Table 2).

**Table 2.** Distribution of primary central nervous system tumors according to histological group and age range, Sergipe, Brazil, 2010-2018.

Age group	Histological groups % (n)					
	Gliomas	Meningiomas	Medulloblastomas	Ependymal tumors	Other astrocytic tumors	Pituitary tumors
0‒14	5.4% (15)	-	45.5% (30)	37.5% (15)	61.2% (30)	-
15‒19	4.7% (13)	1.17% (3)	1.5% (1)	7.5% (3)	4.1% (2)	2.2% (1)
20‒44	25.9% (72)	22.3% (57)	37.9% (25)	42.5% (17)	32.7% (16)	51.1% (23)
45‒54	21.9% (61)	31.0% (79)	9.1% (6)	12.5% (5)	2.0% (1)	17.8% (8)
55‒74	35.9% (100)	34.5% (88)	6.1% (4)	-	-	26.7% (12)
>75	6.1% (17)	11% (28)	-	-	-	2.2% (1)

In the population between 15 and 34 years old, 165 cases were found, which represented 19.2% of the study population. Gliomas occurred most frequently, in 32.7% (n=54) of the cases. In the 35 to 74 age group, which represented 50.4% (n=434) of the population, meningiomas were the most common neoplasms, affecting 47.5% (n=206). The same trend was observed in the sample aged 75 years or over, in which meningiomas were responsible for 61.2% (n=30) of these cases (Table 2).

**Figure 1.**
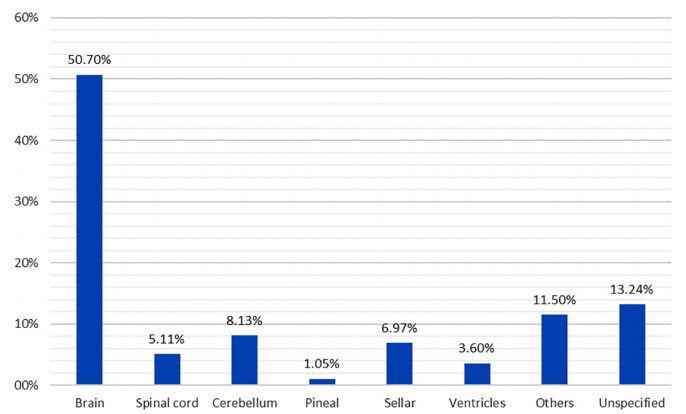
Distribution of primary central nervous system tumors according to the originating site, Sergipe, Brazil, 2010‒2018.

The locations of the tumors were stratified according to the ICD-10 classification. The following locations were found: brain (C70.0, C71.0 to C71.4, C71.8, C71.9), brainstem (C71.7), cerebellum (C71.6), ventricles (C71.5) spinal cord (C70.1, C72.0, C72.1), pineal gland (C75.3, D35.4, D44.5), sella (C75.1, C75.2, D 35.2, D44.3) and unspecified (C70.9, C72.9). Other locations were classified under codes C72.2 to C72.5. The brain was the most common site, followed by the cerebellum (Figure 1). Among the gliomas and meningiomas, the brain was affected in 79.5% (n=221) and 70.6% (n=180) of the cases, respectively. Evaluation of sex and location in relation to meningiomas showed a ratio of 2.4 women for each man, for spinal tumors. Among cerebral tumors, a ratio of 2.3 women for each man with meningioma was found.

In the groups of medulloblastomas and other astrocytic tumors, the cerebellum was the most frequent location, which corresponded to 43.3% (n=26) and 28.6% (n=14) of the cases, respectively. For ependymal tumors, the intraventricular site was the most affected, accounting for 26.2% (n=11) of these neoplasms. The sella site was the location of 100% (n=44) of pituitary adenomas and 50% (n=3) of craniopharyngiomas. The pineal gland was the site of 100% (n=2) of germ cell tumors.

Regarding the WHO grade, the largest proportion of the tumors were classified as grade I, which represented 43.9% (n=378) of the cases, followed by grade IV tumors, corresponding to 27.9% (n=240) of the sample. The entities classified as grade II and grade III represented 14.1% (n=121 and 6% (n=52) of the cases, respectively (Tables 3 and 4).

**Table 3.** Distribution of primary central nervous system tumors according to histology and World Health Organization grade in children (age 0‒14 years), Sergipe, Brazil, 2010‒2018.

Histological group	Grade I		Grade II		Grade III		Grade IV		Unspecified	
	n	%	n	%	n	%	n	%	n	%
Medulloblastomas	-	-	-	-	-	-	26	96.2	1	3.7
Gliomas	-	-	3	20.0	1	6.7	11	73.3	-	-
Other astrocytic tumors	29	100	-	-	-	-	-	-	-	-
Ependymal tumors	-	-	11	78.5	3	21.4	-	-	-	-
Schwannomas and neurofibromas	3	100	-	-	-	-	-	-	-	-
Neuronal and mixed neuronal-glial gliomas	6	85.7	-	-	-	-	-	-	1	14.3
Craniopharyngiomas	-	-	1	100	-	-	-	-	-	-
Tumors of the pineal region	-	-	-	-	-	-	-	-	2	100

**Table 4.** Distribution of primary central nervous system tumors according to histology and World Health Organization graduation in adolescents and adults (age ≥15 years), Sergipe, Brazil, 2010‒2018.

Histological group	Grade I		Grade II		Grade III		Grade IV		Unspecified	
	n	%	n	%	n	%	n	%	n	%
Gliomas	-	-	56	21.4	37	14.1	168	64.1	2	0.76
Meningiomas	218	85.8	29	11.4	5	1.96	-	-	3	1.17
Medulloblastomas	-	-	-	-	-	-	32	96.7	1	3.03
Other astrocytic tumors	17	85.0	1	5.0	-	-	-	-	2	10.0
Ependymal tumors	4	14.3	17	60.7	6	21.4	1	3.6	-	-
Schwannomas and neurofibromas	82	100	-	-	-	-	-	-	-	-
Neuronal and mixed neuronal-glial gliomas	1	8.3	3	25.0	-	-	-	-	8	66.7
Craniopharyngiomas	5	100	-	-	-	-	-	-	-	-
Tumors of the pineal region	-	-	-	-	-	-	-	-	2	100
Hemangioma and hemangioblastomas	13	72.2	-	-	-	-	-	-	5	27.8
Pituitary adenomas	-	-	-	-	-	-	-	-	44	100
Germ cell tumors	-	-	-	-	-	-	-	-	1	100

From the second decade of life onwards, increasing numbers of cases were observed. Moreover, there was a peak between the fourth and sixth decades of life, with subsequent reduction. From 2010 to 2018, the average number of tumors per year was 86.1 (±97.5).

## DISCUSSION

The present study describes the epidemiological aspects of primary CNS tumors diagnosed in Sergipe between 2010 and 2018. The patients’ ages ranged from 6 to 92 years, with a mean of 44.6 years. Patients aged between 45 and 54 years predominated, representing 20.4% (n=176) of the cases. Regarding the epidemiological characterization, predominance of female cases was observed, which corresponded to 56.7% of all the cases. Males accounted for 43.1% of the sample. Although these data from 2010 to 2018 diverged from findings over the previous decade in Sergipe^23^, they were in line with results from other studies^24^^,^^25^. In a previous study in which 2,131 primary CNS tumors were evaluated, 53.7% of the sample was represented by females, while 46.2% were male patients^26^. Similar data were described in the Central Brain Tumor Registry of the United States (CBTRUS). In that series, women comprised 57.9% of all cases of CNS tumors diagnosed in the United States between 2010 and 2014, while men accounted for 42.1%^14^.

In Aracaju, Sergipe, a population-based cancer registry (RCBP) collects high-quality epidemiological data from this state. These data are taken to the National Cancer Institute (INCA) to aggregate with the national estimates for cancer in Brazil. According to INCA data, which are based on what is collected by each state’s RCBP, the estimate for the number of new cases of primary malignant neoplasms of the CNS in Brazil, in 2020, was 11,090 cases. Out of this total, 5,870 were estimated to be among males and 5,220 among females. In Sergipe, 90 new cases were expected in 2020: 40 in males and 50 in females^4^. These estimates are compatible with the data obtained in the present study. However, both benign and malignant tumors were evaluated, which can be explained by the fact that a significant percentage of CNS tumors are not diagnosed through histopathology in Sergipe^27^.

Meningiomas were more prevalent in females than in males, with a ratio of 2.3:1, respectively. This finding is consistent with data from previous studies, which reported that meningeal tumors were approximately three times more frequent among females than among males^19^^,^^25^^,^^28^.

Neuroepithelial tumors comprised approximately half of all the neoplasms observed (50.2%). Among the different histological groups, gliomas had the highest prevalence (32.3%) in the entire sample, followed by meningiomas (29.6%). These findings are consistent with data from other studies in the current literature^26^^,^^29^.

 Regarding age groups, a mean age at the time of diagnosis of 44.64 years was observed. This was lower than what was found in another similar study^30^. The peak prevalence was observed between 45 and 54 years of age, with a subsequent reduction in the number of tumors, especially after the sixth decade of life. These findings are similar to those described in other studies^23^^,^^31^. Higher prevalence of other astrocytic tumors in the age group of 0‒14 years was described in previous studies^12^^,^^14^. Increasing numbers of meningioma cases in older age groups were also described in other studies^14^^,^^32^.

A similar study conducted previously described the epidemiology of CNS tumors in the state of Sergipe between the years 2000 and 2010^23^. In that study, 775 primary CNS tumors were found. Male sex was more prevalent, corresponding to 50.8% (n=429) of the cases. Meningiomas and glioblastomas were the predominant histological types, which represented 21% (n=177) and 18.7% (n=158) of the cases, respectively^23^.

There was an absolute increase in the number of tumors from the previous cohort to the current one, although without statistical significance (p=0.762). We emphasize that some bias exists in this comparison since we cannot confirm that the populations compared were homogeneous or that the methods used in the previous study were similar to those used in the present study. The growth in the number of CNS neoplasms that we observed is a trend corroborated by other studies; however, the evidence is conflicting. In a meta-analysis on 38 articles that was performed in 2014, there were no statistically significant changes in CNS tumor incidence rates^33^. However, in a study based on the Girona cancer registry, an increase in the incidence of CNS neoplasms between 1994 and 2013 was described, consisting of greater numbers of non-malignant tumors^26^. This divergence can possibly be explained in terms of the great heterogeneity of entities among CNS tumors. Moreover, the methodologies and classifications used to study these neoplasms have differed between many of the studies conducted.

In the present study, a progressive increase in the number of tumors diagnosed was found between 2010 and 2018. Other studies have sought to investigate risk factors for possible increased incidence of these tumors^22^^,^^34^^,^^35^^,^^36^. However, increased life expectancy, greater access to diagnostic tests for the population and population growth may influence these findings^18^.

The main limitation of the present study was the significant number of cases that fulfilled the exclusion criteria. These cases were excluded because, in some databases, it was not possible to access immunohistochemical reports to confirm inconclusive histopathological diagnoses. In addition, disparate approaches to the reports between laboratories visited adversely affected the number of examinations available for this study. Studies focusing on the incidence of these tumors are essential in order to determine the evolution of CNS tumors over time.

Nevertheless, our epidemiological description of CNS tumors in the state of Sergipe between 2010 and 2018 is consistent with the data from other recent studies. This characterization also shows similarities with a study conducted on the population of Sergipe between 2000 and 2010 regarding age groups, locations, and most prevalent histological types^25^. The annual average numbers of benign and malignant tumors found were similar, in absolute numbers, to the incidence of malignant CNS tumors estimated for 2020 through the RCBP of Aracaju, Sergipe. This may be related to the low rate of histopathological diagnosis of these tumors in the state of Sergipe^27^. In Brazil, CNS tumor registries do not present the necessary standardization for more reliable data analysis^37^. Therefore, more studies on pathological reports are essential for evaluating the epidemiological evolution of CNS tumors in Sergipe. Studies on the incidence of these tumors and possible associated risk factors are relevant approaches. Knowledge of the evolution profile is vital for optimization of approaches used among the patients affected.
